# Breaking the heterogeneity barrier: a robust prognostic signature for survival stratification and immune profiling in triple-negative breast cancer

**DOI:** 10.3389/fimmu.2025.1611917

**Published:** 2025-09-30

**Authors:** Haixing Shen, Qing Zheng, Zhenyu Wang, Daitian Zheng, Zhenqi Gong, Huaiming Wang, Tianmiao Sun, Jie Pan, Yukai Jin, Xiaohong Zheng, Jingzhi Wang, Jiongjiong Zhang

**Affiliations:** ^1^ Affiliated Cixi Hospital, Wenzhou Medical University, Ningbo, Zhejiang, China; ^2^ Department of Gastrointestinal Surgery, The First Affiliated Hospital of Shantou University Medical College, Shantou, Guangdong, China; ^3^ Second Department of Thyroid and Breast Surgery, The Third Affiliated Hospital of Shenzhen University, Shenzhen, Guangdong, China; ^4^ Cixi Biomedical Research Institute, Wenzhou Medical University, Ningbo, Zhejiang, China; ^5^ Department of Radiotherapy Oncology, The Affiliated Yancheng First Hospital of Nanjing University Medical School; The First People’s Hospital of Yancheng, Yancheng, Jiangsu, China

**Keywords:** immunotherapy, immune infiltration, intra-tumor heterogeneity, prognosis, triple-negative breast cancer, tumor microenvironment

## Abstract

**Background:**

Triple-negative breast cancer (TNBC), a highly heterogeneous breast cancer subtype, poses significant challenges to human health. Intra-tumor heterogeneity (ITH) limits the reliability of conventional prognostic models.

**Methods:**

Using multi-region RNA-seq, we quantified TNBC transcriptomic heterogeneity through an integrative heterogeneity score (IHS). After evaluating inter-patient heterogeneity (IPH) and ITH, prognostic and low-heterogeneity genes were identified and used to build a prognostic risk model with a random survival forest (RSF) algorithm. This model was combined with TNM staging into a nomogram for clinical applicability. We further revealed the distinct immune microenvironment features, somatic mutations, and chemotherapy responses between risk subgroups. Gene expression was validated via RT-qPCR.

**Results:**

Spatial characterization uncovered substantial ITH, evidenced by sharp shifts in PAM50 subtypes and immune infiltration. Two low-heterogeneity biomarkers, CYP4B1 and GBP1, were identified to develop a robust prognostic signature with consistent predictive performance across 3- to 9-year survival endpoints (AUC > 0.6). The high-risk subgroup exhibited reduced immune infiltration, reduced immune checkpoint molecule expression, and poor immunotherapy response rates. Integration of the risk signature with TNM staging created a clinically practical nomogram with superior predictive accuracy (C-index >0.67). Therapeutic vulnerability profiling identified six targeted agents showing increased efficacy in high-risk patients. Dysregulation of signature genes was demonstrated in two TNBC cell lines.

**Conclusions:**

This study established a transcriptomic heterogeneity-resilient prognostic model for TNBC, enabling precise survival stratification and immune microenvironment assessment. The integrative nomogram and risk-guided therapeutic predictions address clinical challenges in TNBC management, advancing personalized treatment strategies.

## 1 Introduction

Triple-negative breast cancer (TNBC), a highly heterogeneous breast cancer subtype, lacks estrogen receptor (ER), progesterone receptor (PR), and human epidermal growth factor receptor 2 (HER2), making it unresponsive to hormonal therapies or targeted treatments. This results in limited clinical therapeutic options and poor prognosis (1). Although TNBC represents only 15%-20% of all breast cancer cases, it exhibits a highly aggressive nature, with patients showing significantly lower five-year survival rates compared to other subtypes, along with elevated risks of local recurrence and distant metastasis (2). Developing accurate prognostic prediction models for TNBC could enable clinicians to perform personalized assessments of disease progression and survival outcomes while optimizing treatment strategies. Currently, conventional histopathology-based prognostic systems still dominate clinical practice. However, histopathological evaluations rely mainly on microscopic morphological observations, which are inherently subjective and prone to variability based on pathologists’ experience. Furthermore, conventional systems typically rely on limited macroscopic indicators such as tumor size and lymph node metastasis, which are insufficient to elucidate molecular mechanisms driving tumor progression or quantify complex immune infiltration and stromal reactions within the tumor microenvironment (TME). Consequently, they fail to distinguish patient subgroups with morphologically similar features but divergent prognostic outcomes.

In recent years, the leapfrog development of high-throughput sequencing technologies has driven continuous innovation in TNBC molecular classification systems and genetic prognostic models. The previous Lehmann classification primarily aimed to achieve molecular-level categorization and identify biological mechanisms, rather than being specifically optimized for precise individual prognosis prediction (3). Although the tumor-infiltrating lymphocytes (TILs) can reflect prognosis, they represent only a portion of the immune microenvironment, failing to directly capture the intrinsic characteristics of tumor cells (4). Compared with traditional prognostic models, transcriptome-based models achieve higher-precision risk stratification and personalized treatment prediction through comprehensive analysis of tumor molecular characteristics and functional states, while revealing therapeutic targets undetectable by conventional morphological methods (5, 6). However, existing studies universally face a critical bottleneck: high intra-tumoral heterogeneity (ITH). This heterogeneity stems not only from immune-stromal interactions within the microenvironment, but also from dynamic evolution of driver mutations or epigenetic dysregulation. Its complexity and dynamic nature significantly undermine the stability and generalization capability of current prognostic models. Even variability in ribonucleic acid (RNA) degradation during sample processing introduces noise signals at the transcriptomic level, causing conventionally identified differentially expressed genes to carry substantial confounding factors rather than true biological signals. While single-cell RNA sequencing (scRNA-seq) and spatial transcriptomics offer new perspectives for resolving cellular subpopulation heterogeneity, their high costs and technical complexity limit applications in large clinical cohorts. Therefore, developing computational frameworks capable of dissociating heterogeneity interference from routine transcriptomic data has become the core challenge for constructing reliable prognostic models.

The multi-region bulk sequencing approach offers technical advantages for studying tumor heterogeneity by enabling the capture of spatial transcriptomic variation features through multi-region sampling and sequencing of tumors. Compared to single-site sampling methods, this technique effectively mitigates sampling bias and more reliably reveals comprehensive transcriptomic characteristics. We analyzed bulk transcriptomic datasets from TNBC patients with multi-region sampling to evaluate how spatial heterogeneity influences biological signal enrichment. Through the evaluation of transcriptomic heterogeneity, we identified spatially stable prognostic biomarkers and constructed a robust TNBC prognostic model resistant to transcriptomic heterogeneity. This model demonstrated consistent long-term prognostic predictive accuracy across The Cancer Genome Atlas (TCGA) and Molecular Taxonomy of Breast Cancer International Consortium (METABRIC) datasets while exhibiting strong immunoinfiltration prediction capability (7, 8). To enhance clinical applicability, we developed a dynamic nomogram integrating transcriptomic profiles with Tumor-Node-Metastasis (TNM) staging, enabling generation of visual risk stratification reports. From a translational medicine perspective, the value of this study lies in establishing a TNBC-specific low-heterogeneity prognostic and immune response prediction system, which will advance innovative clinical practice.

## 2 Methods

### 2.1 Data collection and processing

Multi-region bulk RNA sequencing data of TNBC patients from a previous study was included, encompassing 32 tumor samples from 10 patients (9). This dataset underwent DESeq2 normalization followed by Variance Stabilizing Transformation (VST) to facilitate the identification of low-ITH genes with stable expression patterns (10). The METABRIC TNBC dataset (n = 320) was extracted as the training cohort for prognostic modeling (7). TNBC samples (n = 122) and 113 normal samples from the TCGA breast invasive carcinoma cohort were used for differential expression analysis and served as the prognostic validation cohort (8). The IMvigor210 immunotherapy cohort (n = 348) was utilized for predicting immunotherapy response (11). Somatic mutation data from TNBC cases were obtained from the TCGA database (8). Single-cell dataset GSE148673 (n = 5) (12) and spatial transcriptomic dataset GSE148673 (13) were included in the analysis.

### 2.2 Heterogeneity assessment

Mutant-Allele Tumor Heterogeneity (MATH) was employed to quantify genomic heterogeneity (14). single-region ITH was assessed using the DEPTH2 score (15). A dual-dimensional strategy combining variance analysis and clustering consistency was adopted to evaluate gene heterogeneity in TNBC (16–18). For variance analysis, gene expression data were decomposed using a linear mixed-effects model (via the nlme R package), partitioning variance into within-tumor variance (W) and between-tumor variance (B). The Intra-Tumoral Variability Score (ITVS) was calculated as: ITVS = W/(W + B). This metric (ranging from 0 to 1) reflects the dominance of intra-tumoral heterogeneity when approaching 1. Hierarchical clustering was iteratively performed with the cluster numbers increasing from 1 to the total sample size (N). The patient grouping odds ratio (PGOR) was computed at each clustering level as the proportion of correctly grouped patients. The area under the PGOR curve (AUPC) was quantified via numerical integration, and the clustering consistency score (CCS) was defined as: CCS = 1 − AUPC/(N − 1). Higher CCS (ranging from 0 to 1) indicates lower ITH. The integrated heterogeneity score (IHS) (ranging from 0 to 1) was determined as the geometric mean of ITVS and CCS. A lower IHS correlates with reduced gene-level ITH.

### 2.3 Immune microenvironment and functional analysis

The StromalScore and ImmuneScore were determined through the ESTIMATE (Estimation of STromal and Immune cells in MAlignant Tumors using Expression data) algorithm (19). Additionally, absolute proportions of 22 immune cell subtypes were quantified using CIBERSORT-abs (20). The IOBR package was employed to calculate 15 tumor-intrinsic and 20 TME signatures (21). Functional interpretation was performed through Gene Ontology (GO) and Kyoto Encyclopedia of Genes and Genomes (KEGG) analyses to identify key biological processes (22).

### 2.4 Construction and validation of prognosis model

The limma package was used for the screening of differentially expressed genes (DEGs). Using a false discovery rate (FDR) < 0.05 and a lenient cutoff of |log_2_FC| > 0.137 (equivalent to >10% expression change), this strategy ensures statistical rigor while preserving potential functionally relevant genes. Prognosis-associated DEGs were filtered based on univariate Cox regression and proportional hazards (PH) assumption to build a preliminary gene library. To address transcriptional heterogeneity, protein-coding genes were stratified into low-ITH subgroups using an IHS cut-off of 0.5. Model genes were identified by intersecting low-ITH genes with prognosis-associated DEGs. During model construction, the random survival forest (RSF) algorithm was applied using the METABRIC cohort as the training set. The RSF model was configured with 1000 decision trees and a node size of 36.

### 2.5 Nomogram development

Clinical parameters and molecular features significantly associated with overall survival were selected via univariate Cox regression. Independent prognostic factors were further identified by performing multivariate Cox proportional hazards analysis. For clinical utility, the R package “rms” was used to construct a nomogram prediction model based on independent prognostic factors.

### 2.6 Drug sensitivity prediction

The pRRophetic algorithm was employed to integrate tumor cell line drug screening data with gene expression profiles and to establish a gene expression-drug sensitivity predictive model (23).

### 2.7 Cell culture

The MCF-10A, MCF-10AT, MDA-MB-231, and MDA-MB-453 cell lines (all from ATCC) were used in experiments. Cells were cultured in McCoy’s 5A medium (Gibco) supplemented with 10% fetal bovine serum (Gibco) and 1% penicillin-streptomycin. All cultures were maintained at 37°C with 5% CO_2_ in a standard incubator, with medium replacement every two days.

### 2.8 RT-qPCR

Following total RNA extraction using TRIzol reagent (Invitrogen), reverse transcription was performed with the PrimeScript RT Reagent Kit (TaKaRa). RT-qPCR analysis was conducted with SYBR Green methodology. The thermal cycling protocol comprised: initial denaturation at 95 °C for 30 seconds, followed by 40 amplification cycles (95 °C for 5 seconds and 60 °C for 30 seconds), with subsequent melt curve analysis. Relative gene expression was calculated using the 2^(-ΔΔCt) method and normalized to β-actin as the endogenous control. Three independent biological replicates were implemented. The PCR amplification primer sequences were as follows: CYP4B1, forward: 5’-TGTGCTGAAGCCCTATGTGG-3’, reverse: 5’- CCGGTGTCTCCTCTTCCAAA-3’; GBP1, forward: 5’-AGAGAGGACCCTCGCTCTTA-3’, reverse: 5’- ACATGCCTTTCGTCGTCTCA -3’; β-actin, forward: 5′-TCCATCATGAAGTGTGACGT-3′, reverse: 5-GAGCAATGATCTTGATCTTCAT-3′.

### 2.9 Statistical analysis

Statistical analyses were conducted in R (v4.3.3) and GraphPad Prism 8.0. Non-normally distributed continuous variables were analyzed using Mann-Whitney U or Kruskal-Wallis tests, while categorical variables were assessed via Fisher’s exact test. One-way ANOVA was used to compare means among three or more independent groups. Survival differences were visualized using Kaplan-Meier curves and compared via log-rank test. Cox proportional hazards regression was used to identify prognostic factors, with hazard ratios (HRs) and corresponding 95% confidence intervals (CIs) calculated. For prediction performance evaluation, receiver operating characteristic (ROC) curves were constructed, and the time-dependent discriminative ability of the model was quantified using the area under the curve (AUC). The model’s discrimination was further assessed by the concordance index (C-index). Bootstrap validation with 1000 resampling iterations was performed to evaluate the robustness of the results. Spearman’s rank correlation (nonparametric) and Pearson’s product-moment correlation (parametric) were used for correlation analysis between variables. A significance threshold of *P* < 0.05 was applied unless otherwise specified.

## 3 Results

### 3.1 Genetic and transcriptomic heterogeneity profiles in TNBC

**Figure 1:**
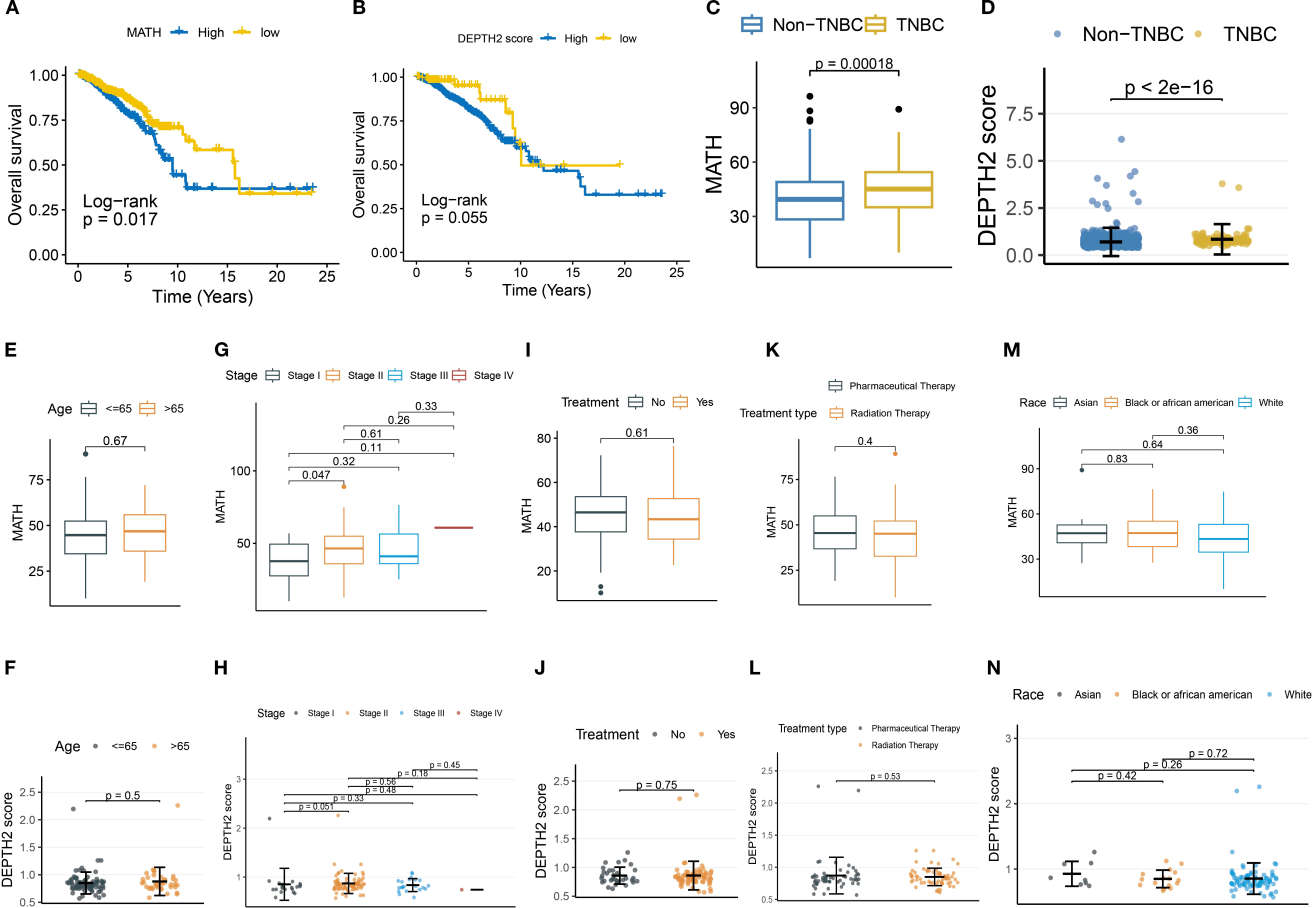
Association of MATH and DEPTH2 scores with clinical parameters. **(A, B)** Kaplan-Meier survival curves for MATH **(A)** and DEPTH2 score **(B)** subgroups. **(C, D)** MATH **(C)** and DEPTH2 scores **(D)** in TNBC versus non-TNBC groups. **(E–N)** MATH and DEPTH2 score distribution in TNBC stratified by clinical features: Age stratification **(E, F)**, tumor stage **(G, H)**, treatment status **(I, J)**, treatment type **(K, L)**, and race **(M, N)**.

First, we used MATH and DEPTH2 scores to quantify ITH at genomic and transcriptomic levels, respectively. Both metrics consistently revealed elevated heterogeneity in TNBC compared to non-TNBC (**Figures 1A, B**; MATH: *P* = 0.00018; DEPTH2: *P* < 2×10⁻¹^6^), aligning with TNBC’s aggressive biology. However, their prognostic utility differed: high MATH significantly predicted poorer overall survival (**Figure 1C**, log-rank *P* = 0.017), while DEPTH2 showed only borderline significance (**Figure 1D**, log-rank *P* = 0.055). Tumor-stage progression impacted both heterogeneity measures similarly. MATH increased significantly from Stage I to Stage II (**Figure 1G**, P = 0.047), plateauing in later stages, potentially due to early clonal expansion followed by stabilization of dominant subclones or cooperative populations in advanced TNBC. The DEPTH2 mirrored this trend, with differences between Stages I and II approaching significance (**Figure 1H**, P = 0.051), suggesting concurrent genomic and transcriptomic heterogeneity shifts during early progression. Notably, neither score varied significantly by age (**Figures 1E, F**), treatment status or type (**Figures 1I–L**), or race (**Figures 1M, N**), indicating that intrinsic tumor biology, rather than extrinsic factors, drives ITH in TNBC. The lack of treatment effect implies conventional therapies may inadequately remodel clonal architecture.

### 3.2 TNBC demonstrates profound IPH and ITH

**Figure 2:**
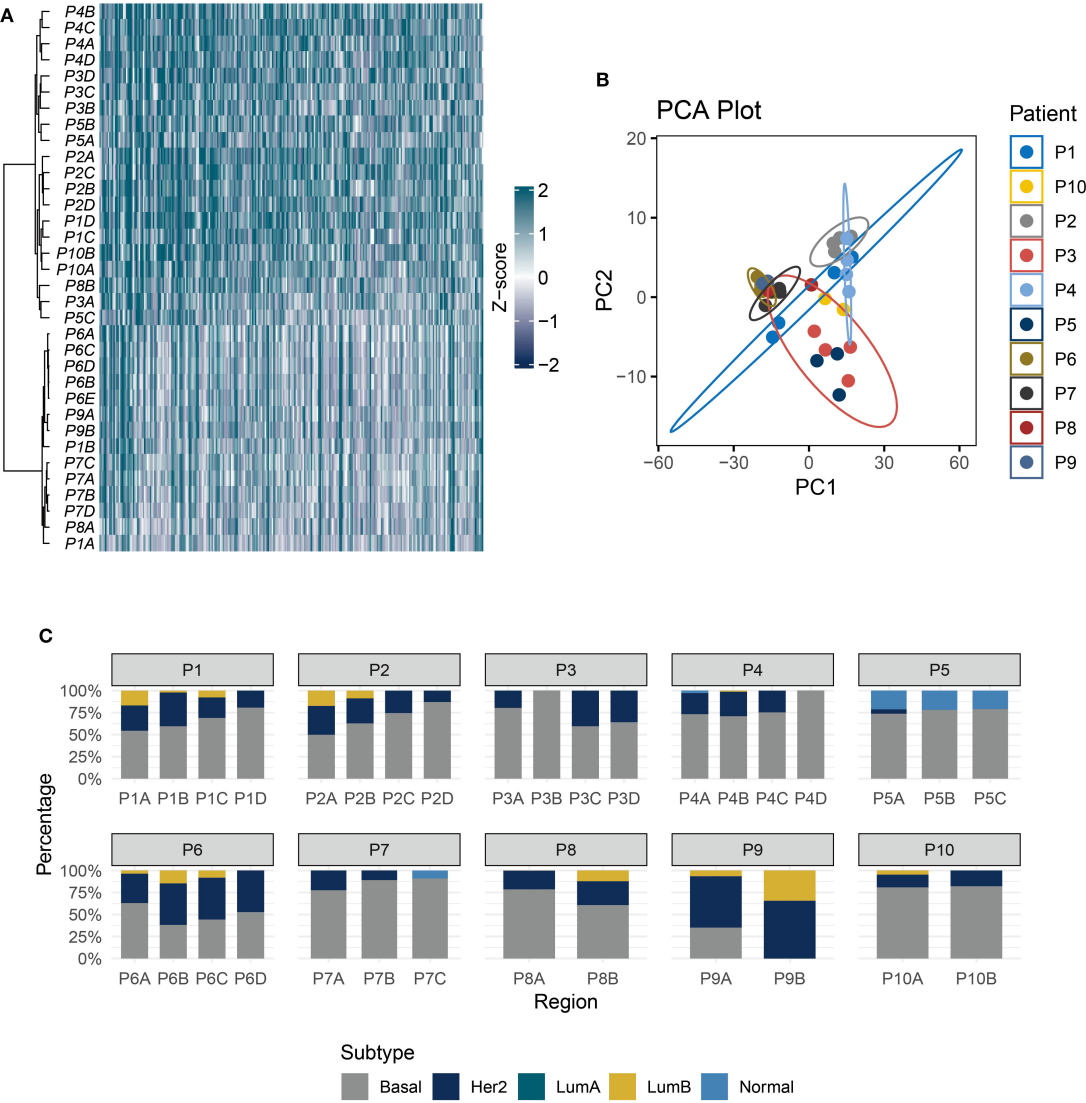
TNBC exhibits strong IPH and ITH. **(A)** The heatmap shows the unsupervised hierarchical clustering of TNBC samples (rows) in the multi-regional cohort according to the top 500 variable expression genes (columns). **(B)** PCA of transcriptome profiles across multi-regional TNBC samples. **(C)** Stacked bar plot showing PAM50 subtype proportions in 10 patients.

Given that single-region ITH estimation is susceptible to sampling bias and may not accurately represent the actual ITH level (24), we further utilized multi-region samples for ITH quantification. At the individual patient level, unsupervised clustering revealed that multiple tumor regions from the same TNBC patient tended to cluster together, highlighting significant inter-patient heterogeneity (IPH) (**Figure 2A**). Principal component analysis (PCA) dimensionality reduction of whole transcriptomic profiles further confirmed that samples from distinct regions exhibit closer clustering associations with patient identity (**Figure 2B**). PAM50 subtyping analysis underscored strong ITH in TNBC (**Figure 2C**). In a representative case (P2), region P2A exhibited mixed features of Luminal B (17.3%), basal-like (49.9%), and HER2-enriched (32.8%) subtypes. In contrast, matched regions (P2C and P2D) displayed sharp increases in basal-like dominance (74.5% and 87.1%, respectively), with the complete loss of the Luminal B subtype.

### 3.3 TME heterogeneity in TNBC

**Figure 3:**
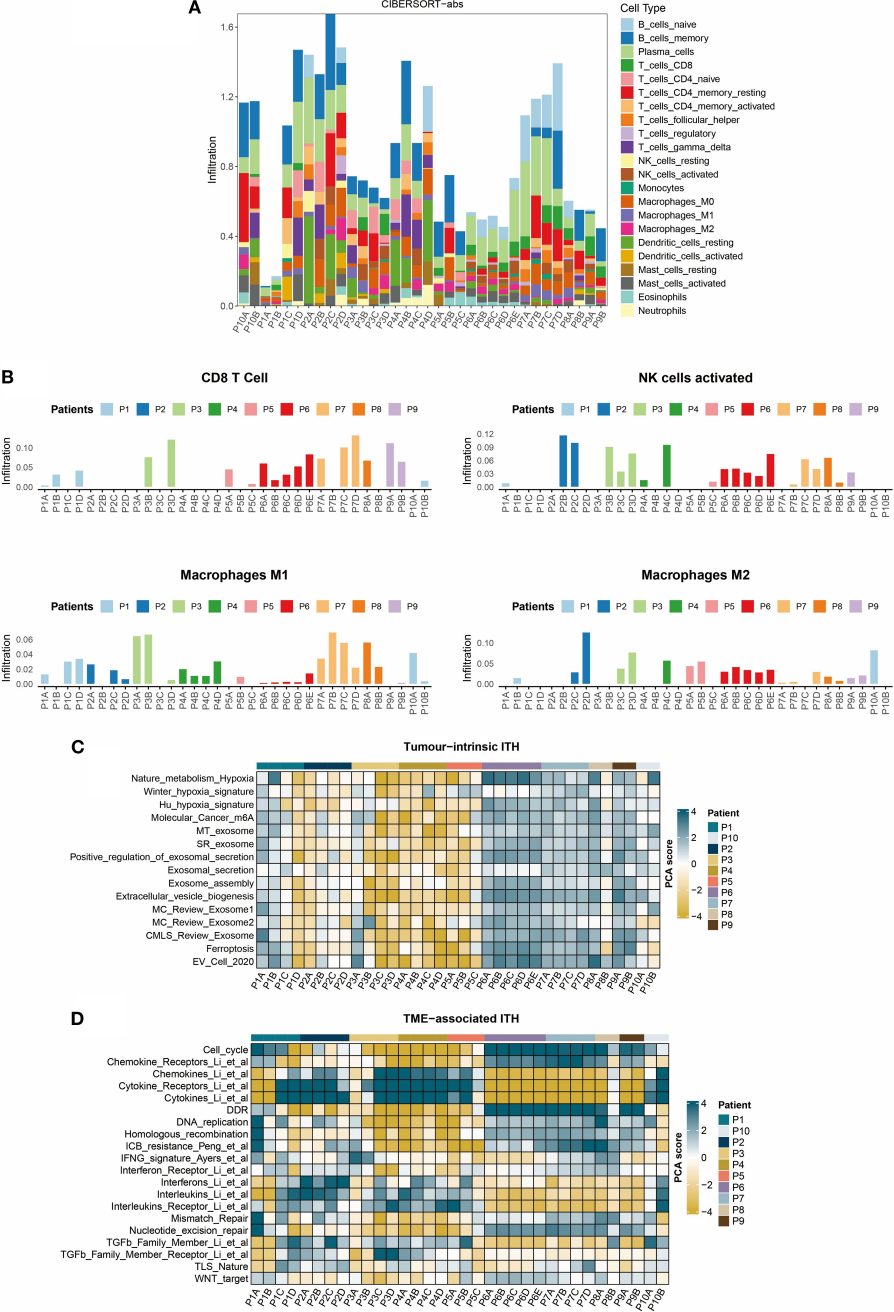
Heterogeneity of the TNBC tumor immune microenvironment. **(A)** Bar plot of immune cell infiltration composition calculated using the CIBERSORT-abs algorithm. **(B)** Regional distribution of key effector cell subsets: Infiltration levels of CD8+ T cells, activated NK cells, and M1/M2 macrophages. **(C)** Heatmap of tumor-intrinsic heterogeneity: Rows represent tumor-intrinsic feature scores and columns denote tumor regions. **(D)** Heatmap of microenvironmental features: Rows include immune signature scores and columns denote tumor regions.

The CIBERSORT-abs algorithm was applied to quantify immune landscape features across 10 multi-region TNBC cases. Results revealed marked fluctuations in immune cell infiltration patterns between patients and across different tumor regions within the same individual (**Figure 3A**). Notably, effector immune cells were not universally detectable, demonstrating pronounced spatial distribution disparities. For instance, CD8+ T cells were highly enriched in region P7D but completely absent in region P7B from the same patient. Similar spatial imbalances were observed in activated NK cells and M1/M2 macrophage subpopulations (**Figure 3B**). Tumor heterogeneity heatmap analysis (**Figure 3C**) uncovered distinct cell death signatures: ferroptosis signaling was activated in region P8A but significantly suppressed in region P8B of the same tumor. This regional variation likely arises from the metabolic reprogramming differences between tumor subclones, where some regions upregulate ACSL4 to promote lipid peroxidation, while others activate GPX4 to sustain antioxidant defenses (25). Additionally, the TME heterogeneity mapping (**Figure 3D**) highlighted that spatially divergent immune pathway activity was downregulated in regions P1A and P1B but strongly activated in P1C and P1D, which reflects region-specific modulation of immune signaling networks.

### 3.4 Gene heterogeneity in multi-region TNBC RNA-seq

**Figure 4:**
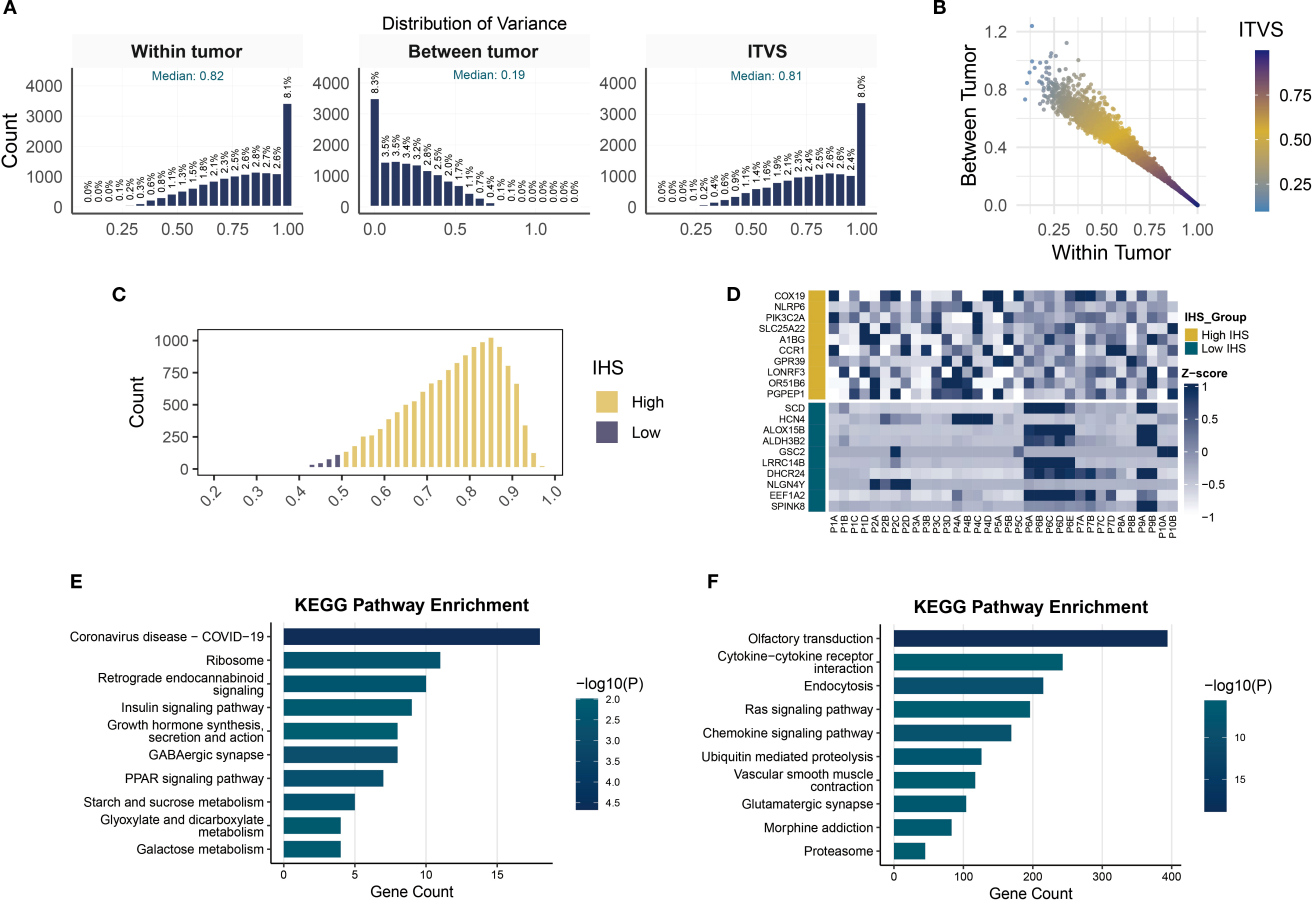
Quantification of gene-level ITH and pathway enrichment analysis. **(A)** Histograms of within-tumor, between-tumor variance and ITVS distribution. **(B)** Scatterplot of gene-level within-tumor/between-tumor variance and ITVS. **(C)** Gene distribution in high- and low-IHS groups. **(D)** Heatmap of expression for top 10 highest and lowest IHS genes. **(E, F)** KEGG pathway enrichment analysis in low- **(E)** and high-IHS **(F)** groups.

We then elucidated the heterogeneity of protein-coding genes in TNBC and its biological implications. **Figure 4A** illustrates the distribution patterns of three variability metrics: within-tumor variance, between-tumor variance, and ITVS. The skewed distribution of within-tumor variance (median 0.82) and ITVS (median 0.81) highlights significant intra-tumor gene heterogeneity in TNBC: 8.1% of genes exhibited extreme within-tumor variance (variance = 1), while 8.3% lacked inter-patient discriminative power (between-tumor variance = 0). Notably, the extreme ITVS = 1 values observed in 8% of genes statistically confirm the multi-clonal coexistence within individual TNBC tumors. The scatter plot in **Figure 4B** further reveals that genes with ITVS < 0.25 are exceedingly rare (only 2 genes), a phenomenon attributed to the combined effect of ITH and IPH in TNBC. For the clustering analysis, we used the CCS approach. The calculation of this score is based on the principle that genes with low ITH should consistently cluster samples from the same patient. An IHS, calculated as the geometric mean of ITVS and CCS (range 0-1), was established, with lower IHS values indicating reduced gene-level heterogeneity. Applying an IHS threshold of 0.5 to 14,071 protein-coding genes, only 2.98% (419 genes) fell into the low-ITH group (**Figure 4C**). A heatmap of the top 10 IHS-ranked genes from each group revealed distinct patterns: low-ITH genes exhibited cross-regional stability, while high-heterogeneity genes displayed pronounced spatial intra-tumor variability (**Figure 4D**). Functional enrichment analysis (**Figures 4E, F**) showed that high-ITH genes were strongly associated with immune-related pathways, including cytokine-receptor interactions and chemokine signaling, suggesting roles in TME reprogramming. Conversely, low-ITH genes were enriched in ribosomal biogenesis and energy metabolism, reflecting conserved roles in core cellular processes. This bifurcation in expression strategies highlights TNBC’s dual survival tactics: maintaining stable expression of metabolic genes for basic cellular functions while preserving heterogeneity in immune-related pathways to enhance adaptability.

### 3.5 DGEs between TNBC and normal breast tissues

**Figure 5:**
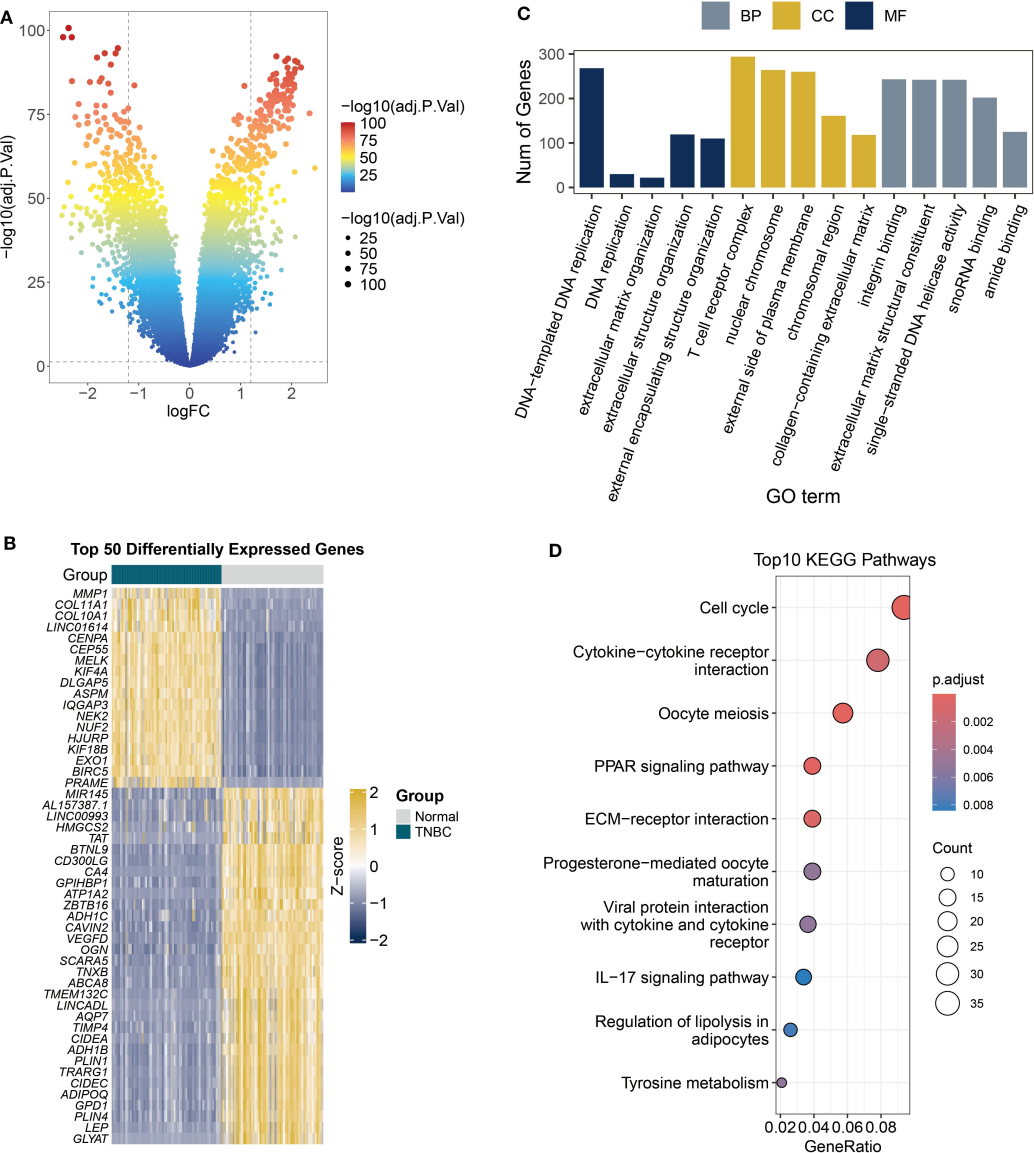
DEGs and functional enrichment in TCGA TNBC samples. **(A)** The volcano plot displays the differential analysis results of tumor versus normal tissues from TCGA TNBC RNA-seq data (FDR < 0.05). **(B)** Heatmap shows the expression patterns of top 50 DEGs. **(C)** Analysis of the biological roles of DEGs (|logFC| > 1, adjusted *P* < 0.05) using GO terms from biological processes (BP), cellular components (CC), and molecular functions (MF). **(D)** The KEGG bubble plot displays the top 10 enriched pathways of DEGs (|logFC| > 1, adjusted *P* < 0.05).

Tumor-associated DEGs hold particular biological significance compared to those in healthy tissue, as they may reveal molecular-level dysregulation mechanisms driving tumorigenesis, development, and malignant phenotypes (26). DEGs are more likely to affect patient survival rates. This study compared gene expression profiles between TNBC and normal breast tissues using transcriptomic data from the TCGA database (**Figure 5A**). We identified 18,252 significantly DEGs based on screening criteria (|log2FC| > 0.137, FDR < 0.05). Heatmap visualization of the top 50 most significant DEGs revealed distinct clustering patterns in the TNBC group (**Figure 5B**), including upregulated MMP1 and COL1A1. The elevated expression of these genes points to their potential contribution to tumor invasion and metastasis via extracellular matrix remodeling (27). GO enrichment analysis revealed associations with cell proliferation and cycle regulation, including terms such as DNA replication and chromosomal region (**Figure 5C**). KEGG pathway analysis identified enrichment of cell cycle-related genes and ECM-receptor interaction pathways (**Figure 5D**), which may mediate the invasive potential of tumors.

### 3.6 Construction and validation of a low-ITH prognostic model

**Figure 6:**
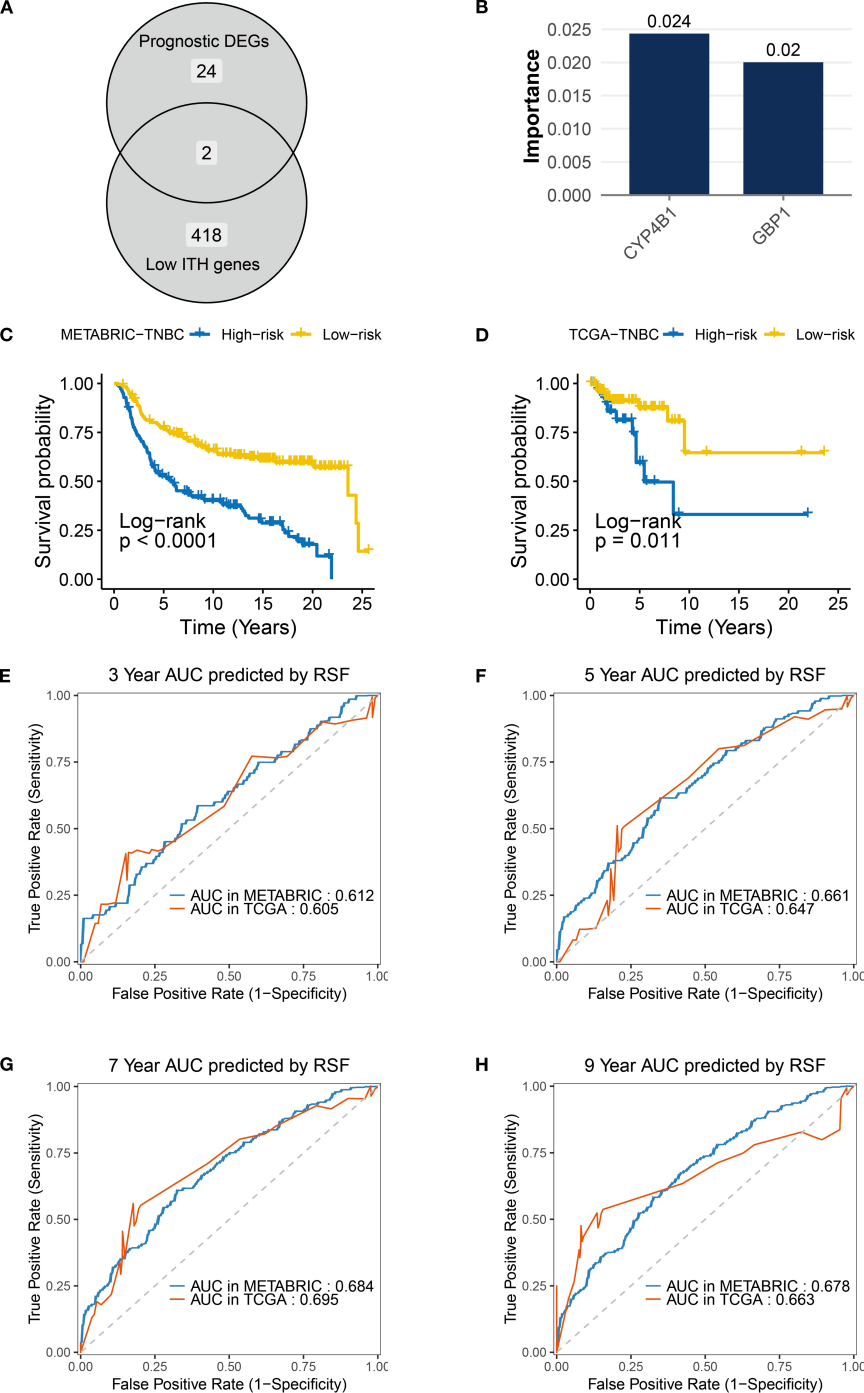
Construction and validation of the low-ITH prognostic model in TNBC. **(A)** Venn diagram indicating overlap between prognostic DEGs (n = 24) and low-ITH genes (n=418). **(B)** RSF importance scores for model genes CYP4B1 (0.024) and GBP1 (0.02). **(C, D)** Survival curves for risk groups in TNBC samples from METABRIC and TCGA datasets. **(E–H)** ROC curves showing AUC values for RSF model predicting 3-/5-/7-/9-year survival in TNBC samples from METABRIC and TCGA datasets.

By analyzing transcriptomic heterogeneity, this study established a prognostic stratification model for TNBC patients with low ITH. To address cohort heterogeneity, we selected genes with consistent HR directions in the TCGA and METABRIC datasets. Venn diagram analysis of 418 low-ITH genes (IHS: 0-0.5) and 24 prognosis-related differentially expressed genes (**Figure 6A**) identified two core regulators, CYP4B1 and GBP1 (Both genes met the Cox model’s PH assumption; **Supplementary Figure S1**). RSF algorithm assessed gene importance, assigning weight scores of 0.024 and 0.02 to CYP4B1 and GBP1, respectively (**Figure 6B**). A risk prediction model was constructed: Patient risk score = (0.024 × CYP4B1 expression) + (0.02 × GBP1 expression). In the METABRIC cohort, TNBC patients in the high-risk group exhibited significantly shorter overall survival compared to the low-risk group, a finding validated in the TCGA-TNBC dataset (**Figures 6C, D**). Time-dependent ROC curve analysis demonstrated robust predictive performance in METABRIC (3-year: 0.612, 5-year: 0.661, 7-year: 0.684, 9-year: 0.678) and TCGA validation (3-year: 0.605, 5-year: 0.647, 7-year: 0.695, 9-year: 0.663), demonstrating cross-platform applicability of the model (**Figures 6E–H**).

### 3.7 Development of a nomogram integrating risk score and TNM stage

**Figure 7:**
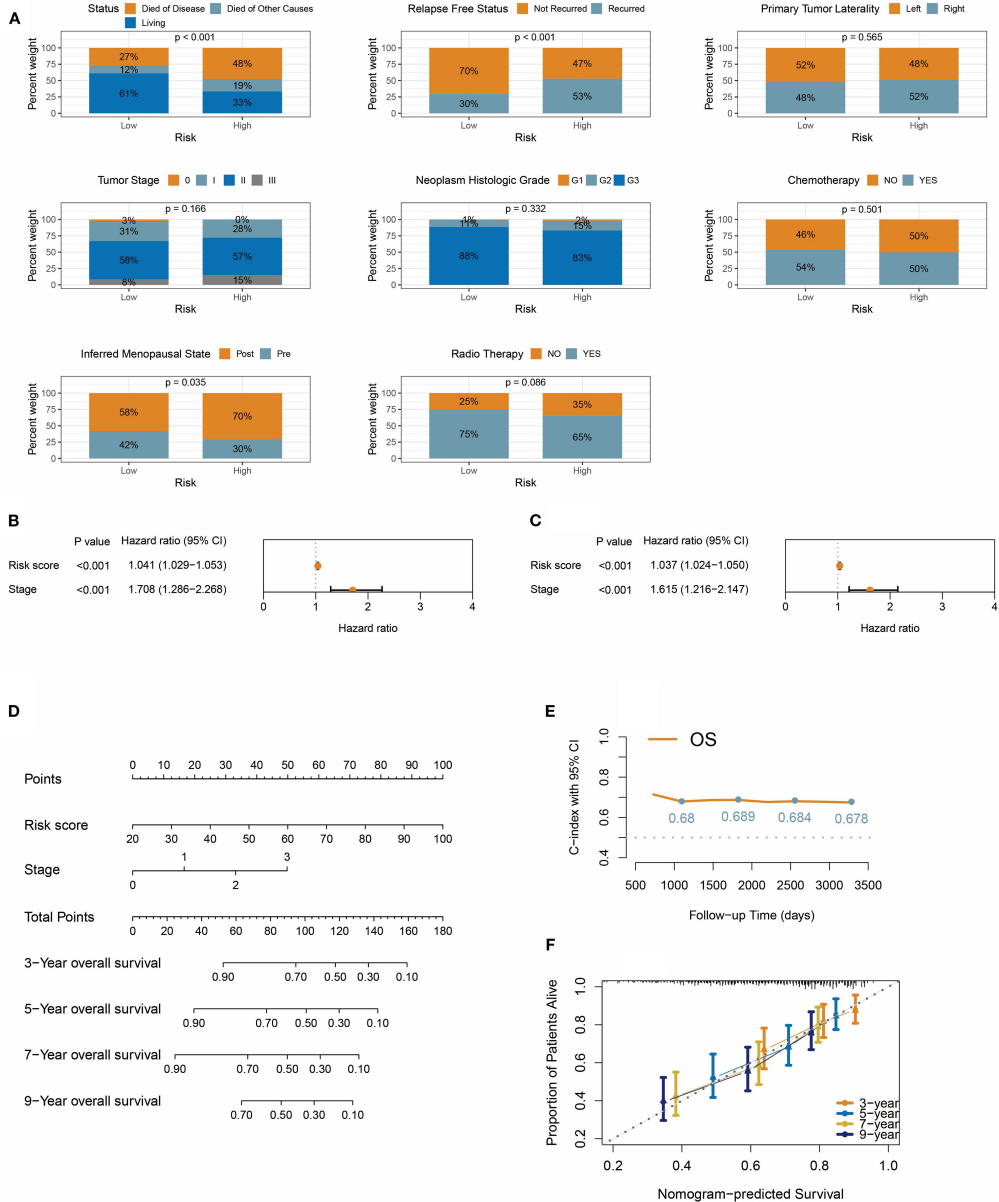
Integrative prognostic nomogram combining risk score and TNM stage. **(A)** Distribution of survival status, recurrence, tumor laterality, stage, histologic grade, treatment, and menopausal status in risk groups. **(B, C)** Univariate forest plot **(B)** and multivariate forest plot **(C)** demonstrate the impact of risk score on overall survival. **(D)** Nomogram integrates risk score and TNM stage for predicting overall survival in TNBC patients. **(E)** Time-dependent C-index for nomogram at 3/5/7/9 years. **(F)** The calibration curve shows that the predicted probabilities from the nomogram have high consistency with the actual observed probabilities.

Analysis of TNBC patient data in the METABRIC cohort revealed that the risk score stratification effectively distinguished subgroups with significant prognostic differences: 48% of TNBC-related deaths occurred in the high-risk group (vs. 27% in the low-risk group), and recurrence rates were elevated in high-risk patients (**Figure 7A**). Notably, while no statistically significant differences were observed between the two groups in TNM staging, histological grade, primary tumor laterality, or treatment history (radiotherapy/chemotherapy), the proportion of postmenopausal patients was significantly higher in the high-risk group (70% vs. 58%, *P* = 0.035), suggesting that hormonal status may influence prognosis through non-canonical mechanisms (**Figure 7A**).

We then evaluated the prognostic predictive power of the risk score, TNM stage, and pathological grade. Univariate Cox regression analysis identified significant associations between overall survival and the risk score (HR = 1.041, 95% CI: 1.029-1.053) and clinical stage (HR = 1.708, 95% CI: 1.286-2.268), both with p<0.001 (**Figure 7B**). However, pathological grade was not statistically significant in univariate Cox analysis. Crucially, in the multivariate Cox model, the risk score and clinical stage retained independent prognostic value (**Figure 7C**), indicating their synergistic predictive utility. Based on these findings, a nomogram integrating the risk score and clinical stage was developed (**Figure 7D**). This visual tool quantifies the contribution weight of each variable, enabling clinicians to rapidly calculate 3-, 5-, 7-, and 9-year overall survival probabilities. Model validation demonstrated robust performance, with the C-index consistently exceeding 0.67 during the 3- to 9-year follow-up period (**Figure 7E**), confirming sustained predictive accuracy over time. Time-calibration curves (**Figure 7F**) further validated model precision, showing strong alignment between predicted and observed survival rates at 3, 5, 7, and 9 years.

### 3.8 Immune infiltration in risk subgroups

**Figure 8:**
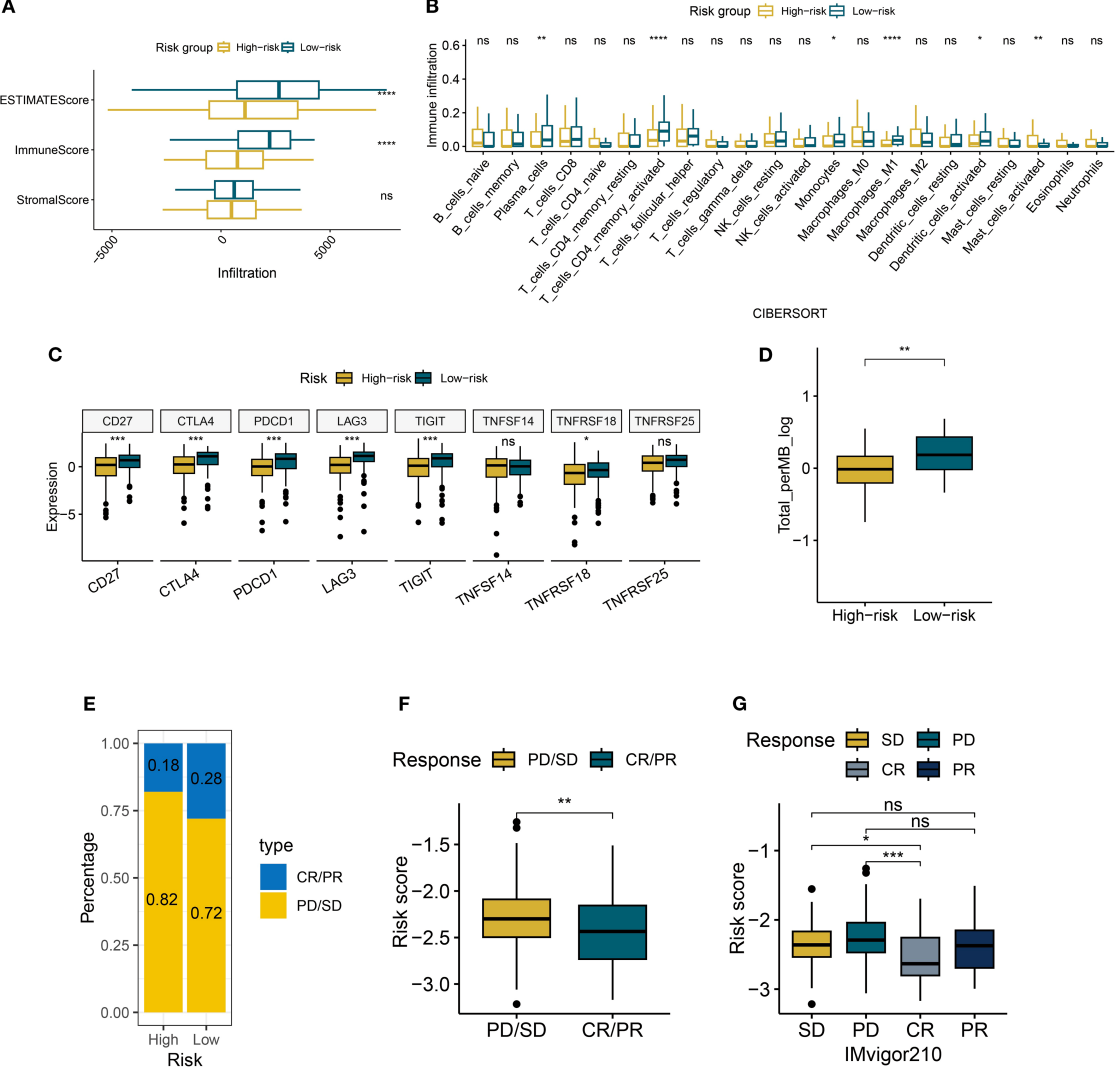
Immune characteristics of risk groups. **(A)** Boxplots of ESTIMATE/immune/stromal scores in risk subgroups. **(B)** The boxplot illustrates the differences in immune cells between risk groups, as calculated by CIBERSORT. **(C)** Boxplots of immune checkpoint expression between risk groups. **(D)** Boxplots of tumor mutational burden (TMB) between risk groups. **(E)** Bar plot of clinical response rates between risk groups (CR, complete response; PR, partial response; SD, stable disease; PD, disease progression). **(F, G)** Boxplots of risk scores across treatment response subgroups. *P < 0.05, **P < 0.01, ***P < 0.001, ****P < 0.0001, ns, not significant.

This study revealed that high-risk patients exhibit an immune-suppressive state compared to low-risk patients. Using the ESTIMATE algorithm, we found that the ESTIMATEScore and ImmuneScore were significantly lower in the high-risk group compared to the low-risk group, while the StromalScore showed no statistical difference (**Figure 8A**). This suggests that the reduced immune activity in high-risk patients primarily arises from altered immune cell composition rather than stromal components. Further immune infiltration quantification via the CIBERSORT algorithm (**Figure 8B**) showed reduced infiltration levels of anti-tumor immune subpopulations in the high-risk group. Specifically, M1 macrophages, dendritic cells, plasma cells, monocytes, and CD4+ memory T cells were markedly decreased (all *P* < 0.05). Notably, CD8+ T cells, natural killer cells, M2 macrophages, and regulatory T cells showed no statistically significant differences.

Analysis of immune checkpoint expression profiles (**Figure 8C**) revealed significant downregulation of key regulators (CD27, CTLA4, and PDCD1) in the high-risk group. However, the expression of TNFSF14 and TNFRSF25 were not statistically significant, highlighting heterogeneity in checkpoint regulation. Concurrently, the reduced tumor mutational burden (TMB) in high-risk patients may further impair antigen presentation efficiency (**Figure 8D**). In the IMvigor210 cohort, we observed increased resistance to immune checkpoint blockade therapy in the high-risk group. The combined proportion of progressive disease (PD) and stable disease (SD) cases reached 82% in the high-risk group, surpassing the 72% in the low-risk group (**Figure 8E**). Patients who achieved complete or partial responses (CR/PR) had significantly lower median risk scores than those with PD/SD (**Figures 8F, G**). These findings collectively suggest that the risk scoring system effectively identifies immunotherapy non-responders, offering critical insights for clinical decision-making.

### 3.9 Mutational landscape of risk subgroups

**Figure 9:**
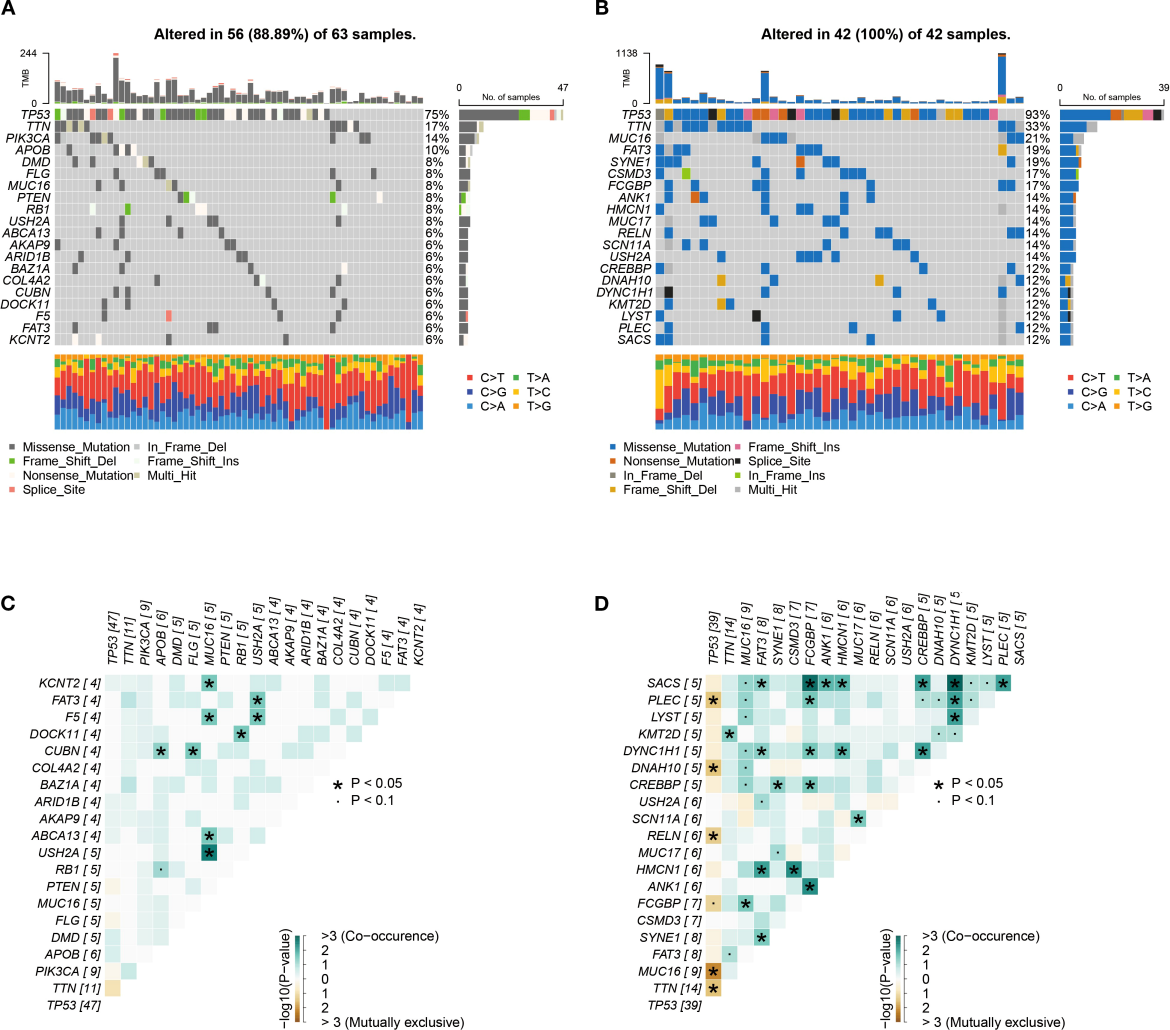
Genomic alteration profiles. **(A, B)** High-frequency mutated genes in 105 TNBC samples in high-risk group **(A)** and low-risk group **(B)**. **(C, D)** Co-occurrence and mutual exclusivity heatmaps of top 20 genes in high-risk group **(C)** and low-risk group **(D)**.

This study integrated genomic data from 105 TCGA TNBC tumor samples (63 high-risk, 42 low-risk) to characterize mutation patterns and risk interactions. **Figure 9A** shows that 56 out of 63 high-risk samples (88.89%) harbored at least one genetic alteration, with TP53 mutations being the most frequent (75%), followed by TTN (17%) and PIK3CA (14%), dominated by missense mutations. All low-risk samples displayed genetic alterations, with TP53 mutations occurred in 93% of cases, alongside high-frequency mutations in TTN (33%) and MUC16 (21%) (**Figure 9B**). Co-occurrence and exclusivity analysis (**Figures 9C, D**) revealed significant associations: USH2A and MUC16 exhibited strong co-occurrence (*P* < 0.05), suggesting synergistic oncogenic pathways, while TP53 and MUC16 displayed mutual exclusivity.

### 3.10 Drug sensitivity screening for high-risk patients

**Figure 10:**
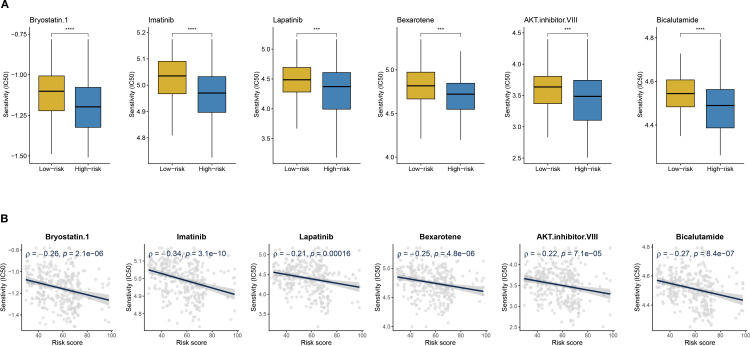
Drug sensitivity and risk score. **(A)** Boxplots of IC50 values for six drugs in low-risk and high-risk groups. **(B)** The scatter plot shows the correlation between IC50 and risk score. ***P < 0.001, ****P < 0.0001.

Given the poor prognosis and immunotherapy resistance in high-risk patients, the pRRophetic platform identified six anticancer agents with significant negative correlations to risk scores (R < −0.02) and lower IC50 values in the high-risk group: Bryostatin.1, AKT inhibitor VIII, Imatinib, Bexarotene, Lapatinib, and Bicalutamide (**Figures 10A, B**). These findings suggest that risk score-guided drug selection may optimize personalized therapy for high-risk patients.

### 3.11 Expression patterns of CYP4B1 and GBP1 in TNBC

**Figure 11:**
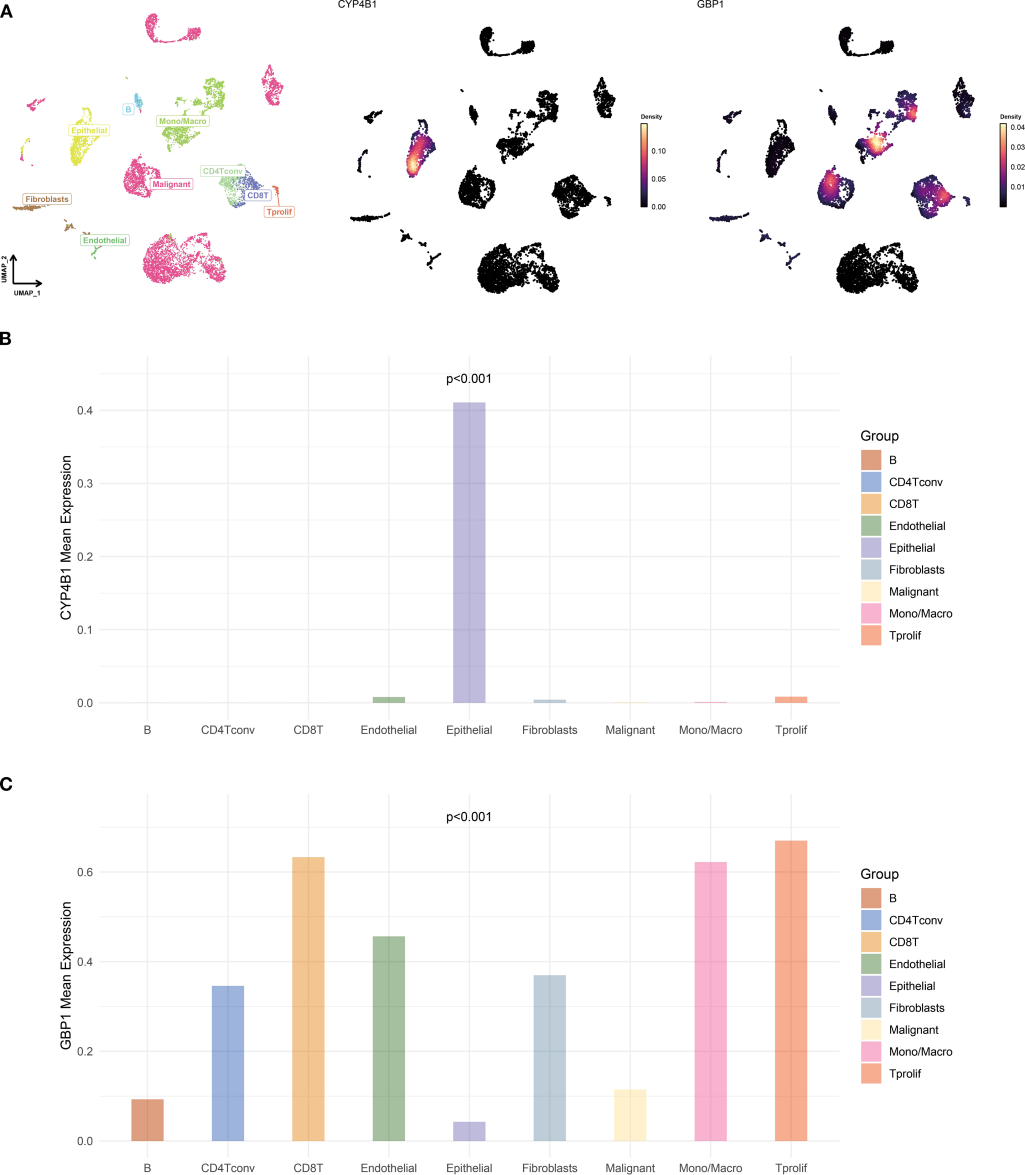
Single-cell atlas and feature gene expression. **(A)** UMAP visualization of cell types and gene expression patterns in GSE148673 dataset. **(B, C)** Bar charts showing the expression of CYP4B1 **(B)** and GBP1 **(C)** in different cells.

Single-cell and spatial transcriptomic analyses of TNBC tissues were conducted through web-based platforms (https://grswsci.top/). Single-cell sequencing resolved nine distinct functionally-defined cell clusters (**Figure 11A**), with the epithelial subpopulation demonstrating marked CYP4B1 enrichment (**Figure 11B**). Further gene expression profiling (**Figure 11C**) revealed that GBP1 exhibited significantly elevated expression levels in malignant cell populations compared to normal epithelial cells.

**Figure 12:**
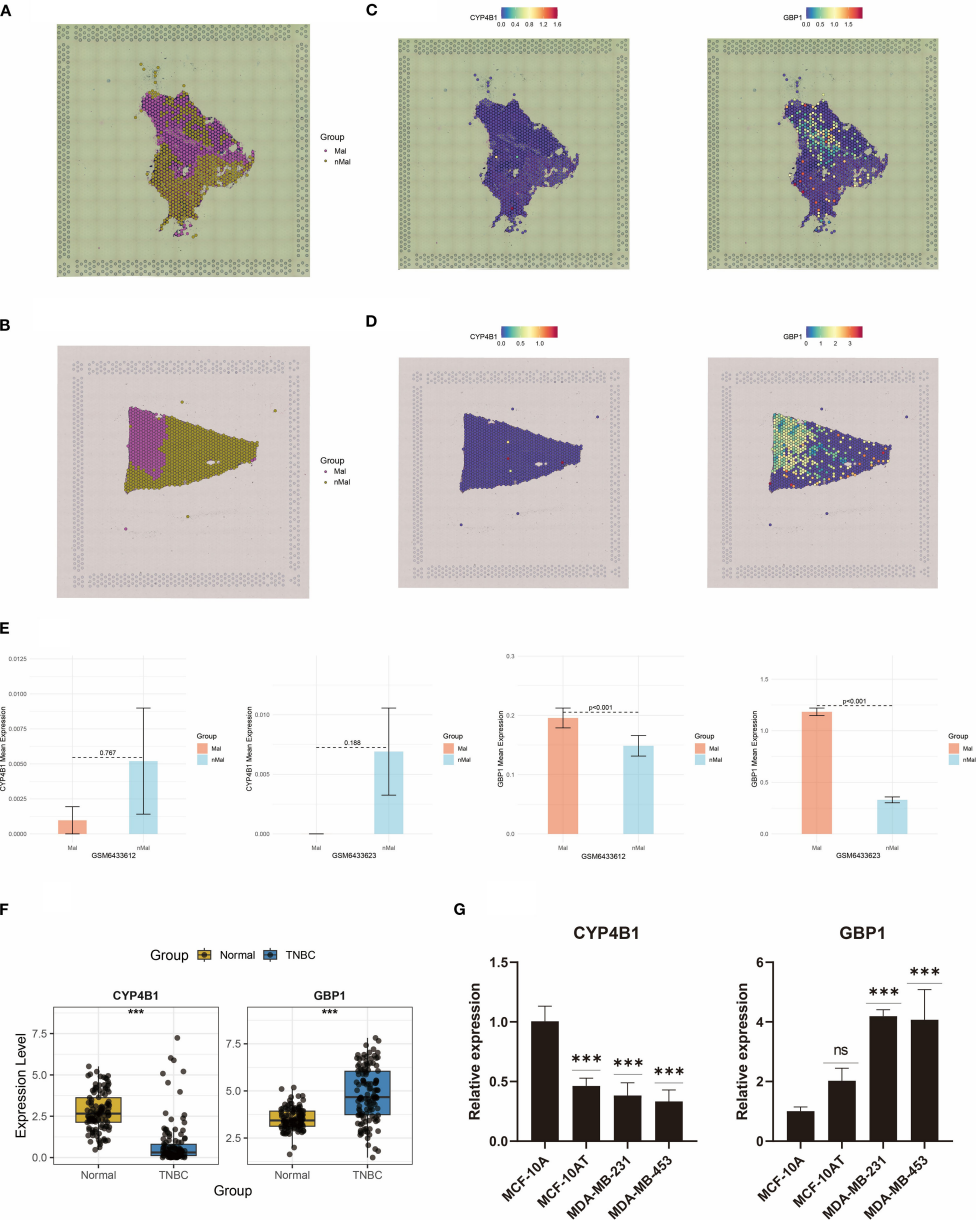
Spatial transcriptomics and RT-qPCR analyses reveal dysregulated expression of CYP4B1 and GBP1. **(A, B)** Spatial demarcation of malignant and non-malignant regions. **(C, D)** Spatial expression patterns of CYP4B1 and GBP1. **(E)** Bar graph quantifying CYP4B1 and GBP1 expression levels between malignant and non-malignant regions. **(F)** Dysregulated expression of CYP4B1 and GBP1 in TCGA TNBC cases. **(G)** RT-qPCR validation of CYP4B1 and GBP1 expression dysregulation in two TNBC cell lines. ***P < 0.001, ns, not significant.

Spatial transcriptomics was performed on two representative TNBC samples from the GSE210616 dataset. Spatial regionalization delineated tissue areas containing malignant cells (proportion > 0) as malignant regions, while regions exclusively composed of normal cells were designated as normal areas (**Figures 12A, B**). CYP4B1 exhibited significantly elevated expression in non-malignant regions, whereas the GBP1 displayed specific enrichment in tumor malignant zones (**Figures 12C, D**). Barplots validated this heterogeneous expression pattern for both genes across TNBC specimens (**Figure 12E**).

Boxplot analysis revealed dysregulated expression of CYP4B1 and GBP1 in TCGA-derived TNBC samples (**Figure 12F**). To corroborate these findings, RT-qPCR validation of CYP4B1 and GBP1 expression was conducted (**Figure 12G**). Furthermore, proteomic data from TCGA breast cancer also validated the expression trends of CYP4B1 and GBP1 (**Supplementary Figure S2**). Compared with normal mammary MCF-10A cells, CYP4B1 showed marked downregulation in premalignant MCF-10AT cells and aggressive TNBC cell models (MDA-MB-231 and MDA-MB-453). Notably, GBP1 demonstrated pronounced upregulation in both malignant cell lines.

## 4 Discussion

This study revealed the complex characteristics of ITH in TNBC and its impact on clinical outcomes. Our findings not only deepen the understanding of TNBC’s biological behavior but also offer new perspectives for developing precision medicine strategies.

The stage-specific evolution of genetic heterogeneity in TNBC was confirmed in this study. MATH scores showed significant increasing trends in stages I-II, while no marked differences were observed in stages III-IV. This phenomenon could be attributed to limited sample size on one hand and aligns with the “bottleneck effect” model of tumor evolution proposed by Sottoriva et al. (28) on the other. Elevated genetic heterogeneity in early-stage tumors may reflect Darwinian selection processes under microenvironmental pressure, whereas ITH tends to stabilize when dominant clones take over. Notably, conventional therapeutic approaches failed to significantly alter MATH scores, suggesting that cytotoxic agents or radiotherapy primarily eliminates sensitive clones while inadequately suppressing adaptive evolution of subclones (29). The DEPTH2 scores at the transcriptome level demonstrated clinical patterns similar to those of MATH, yet showed slightly weaker prognostic discrimination. This phenomenon may stem from a decoupling mechanism between genomic mutations and gene expression regulation (30). Some driver mutations may be buffered at the RNA expression level, potentially explaining why genetic heterogeneity carries greater prognostic significance. However, the highly significant differences in DEPTH2 scores between TNBC and non-TNBC groups highlight its potential value as a supplementary marker for TNBC molecular subtyping, warranting further validation through expanded sample studies.

Using multi-regional TNBC samples, this study revealed the spatial hierarchy of tumor heterogeneity. The dramatic shifts in PAM50 subtypes across tumor regions may represent the dominance of evolutionarily selected “fittest phenotypes” within specific microenvironments. In immune landscape analysis, CD8+ T cells exhibited an unbalanced distribution pattern, which could be explained by aberrant tumor vascularization affecting immune cell chemotaxis and subclone-specific antigen variation driving localized immune editing (31, 32). Additionally, heterogeneous ferroptosis pathway activity suggests that hypoxia gradients might regulate ACSL4/GPX4 balance via HIF-1α, creating regional divergences in ferroptosis susceptibility (33).

Given the high ITH in TNBC and the vulnerability of conventional biomarkers to ITH-induced predictive instability, this study proposed a “low-ITH gene priority” modeling strategy. We successfully identified CYP4B1 and GBP1 as core biomarkers with IHS < 0.5 and significant prognostic relevance. Compared to high-ITH genes (IHS > 0.5), these genes demonstrated cross-regionally stable expression patterns, maintaining reliable detection across spatiotemporal sampling conditions. Mechanistically, CYP4B1 (a cytochrome P450 family member) may mediate metabolic processes in the TME (34), while GBP1 (an interferon-induced protein) participates in cancer cell growth and promotes metastasis in TNBC (35). Minimal overlap was observed between low-ITH and differentially expressed prognostic genes (only 2/419), underscoring the necessity of combining heterogeneity screening for precise biomarker identification. The prognostic risk model achieved robust predictive performance in METABRIC and TCGA cohorts (3-, 5-, 7-, and 9-year AUC > 0.6), validating the feasibility of low-ITH genes for outcome prediction. This model overcomes TNM staging limitations by stratifying patients with identical TNM classifications into prognostically distinct subgroups. Multivariate Cox regression confirmed the risk score’s independent prognostic value, while its combination with TNM staging in a nomogram (C-index > 0.67), providing a clinical decision-making framework.

The immune cell infiltration profile revealed the biological basis of risk stratification, with high-risk groups exhibited exhaustion of anti-tumor immune responses. Specifically, tumor-associated immune cells displayed marked phenotypic polarization imbalance, with significantly reduced proportions of pro-inflammatory M1 macrophages. Further analysis uncovered atypical checkpoint regulation patterns in immune evasion among high-risk patients. Unlike the classical “cold tumor” model characterized by PD-L1/CTLA4 overexpression (36), this subgroup showed concurrent downregulation of checkpoint molecules (CTLA4, PDCD1), suggesting aberrant activation of specific immune-exhausted subtypes. Data from the IMvigor210 cohort supported this observation, showing reduced immunotherapy response rates in high-risk patients, indicating that the risk score could serve as a novel predictive biomarker for immunotherapy resistance.

## 5 Conclusion

This study systematically evaluates the multidimensional features of TNBC heterogeneity. By prioritizing low-ITH genes, we developed a prognostic model with temporal stability, uncovering intrinsic links between risk scores and immune microenvironment remodeling. The low-ITH model and nomogram provide clinically applicable tools for TNBC outcome prediction and personalized treatment. These findings deepen the understanding of TNBC heterogeneity and establish novel frameworks for precision subtyping in TNBC.

## Data Availability

The original contributions presented in the study are included in the article/**Supplementary Material**. Further inquiries can be directed to the corresponding authors.

## Supplementary material

Supplementary Figure 1
**(A, B)** The Cox model’s PH assumption of CYP4B1 **(A)** and GBP1 **(B)**.

Supplementary Figure 2
**(A, B)** The density plots display the protein expression levels of CYP4B1 **(A)** and GBP1 **(B)** in the normal group and the breast cancer group from TCGA database.
