# Supplementary figures and images for: Breaking the heterogeneity barrier: a robust prognostic signature for survival stratification and immune profiling in triple-negative breast cancer
Source: Front Immunol. 2025 Sep 30;16:1611917. doi: 10.3389/fimmu.2025.1611917 (PMC12518079; doi:10.3389/fimmu.2025.1611917)

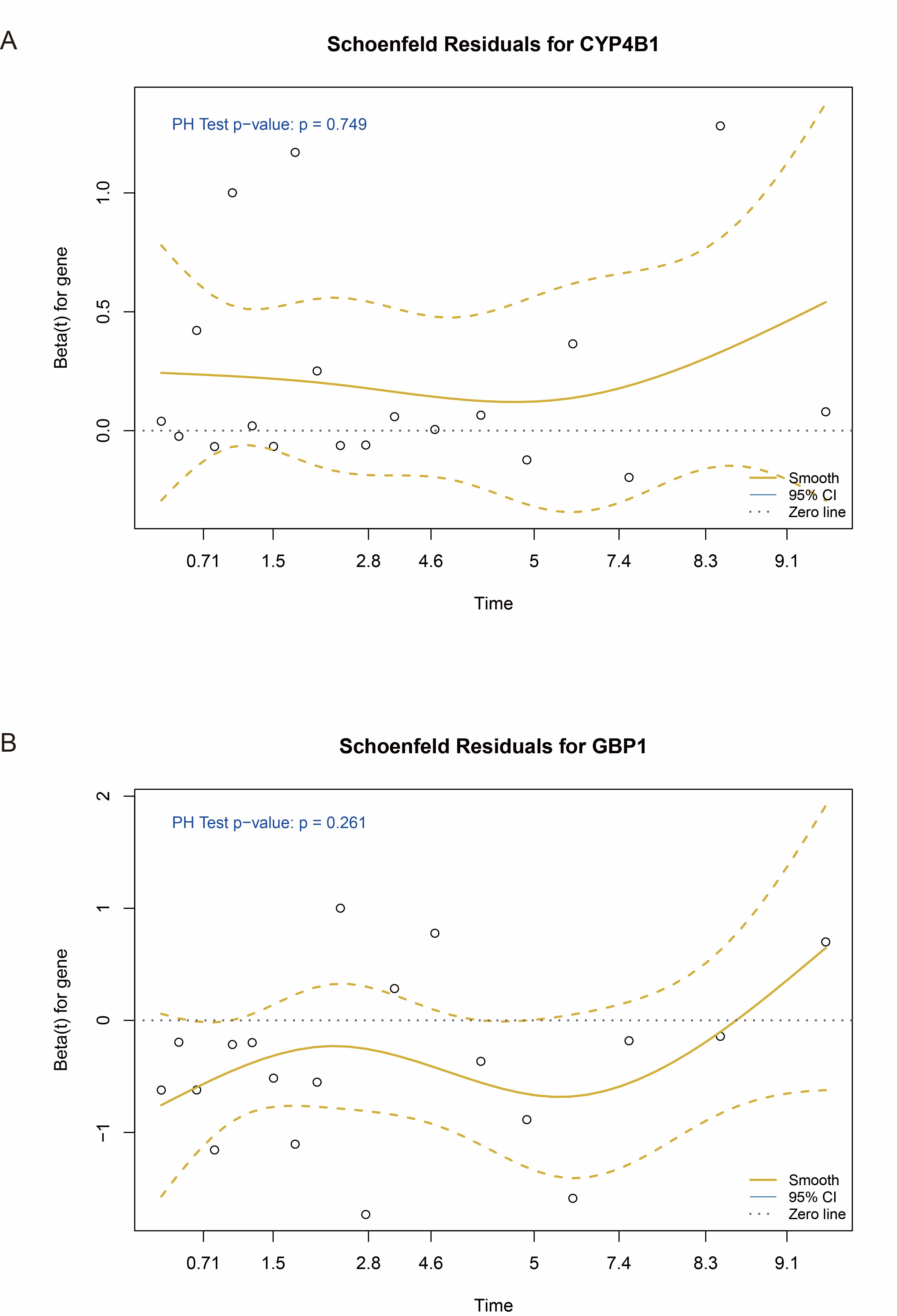

Supplement: Supplementary Figure 1 — (A, B) The Cox model’s PH assumption of CYP4B1 (A) and GBP1 (B). [file Image1.jpeg]

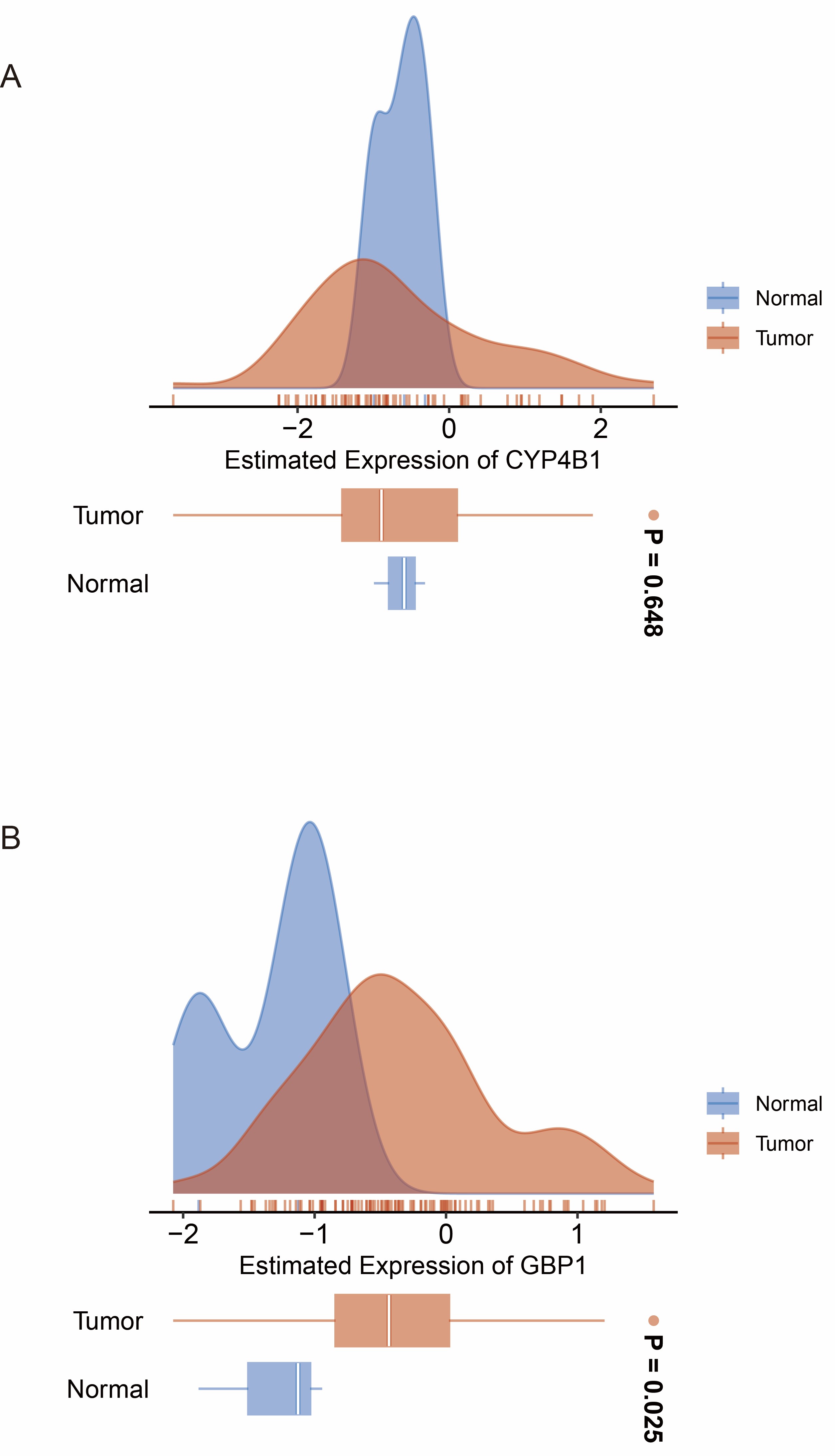

Supplement: Supplementary Figure 2 — (A, B) The density plots display the protein expression levels of CYP4B1 (A) and GBP1 (B) in the normal group and the breast cancer group from TCGA database. [file Image2.jpeg]
